# The Value of a Peer Mentorship Programme for Postgraduate Students in New Zealand: A Qualitative Study

**DOI:** 10.1007/s40670-024-02189-4

**Published:** 2024-10-12

**Authors:** Joseph C. C. Chen, Julia R. Plank, Angela Tsai, Mataroria Lyndon, Marcus A. Henning

**Affiliations:** 1 School of Pharmacy, Faculty of Medical and Health Sciences, University of Auckland, Auckland, New Zealand; 2 Department of Anatomy and Medical Imaging, Faculty of Medical and Health Sciences, University of Auckland, Auckland, New Zealand; 3 Centre for Medical and Health Sciences Education, Faculty of Medical and Health Sciences, University of Auckland, Auckland, New Zealand

**Keywords:** Postgraduate students, Wellbeing, Social networking, Pastoral care, Peer mentoring, Higher education

## Abstract

Postgraduate students face significant challenges such as feelings of isolation, lack of guidance, and negotiating multiple identities. Navigating these challenges can adversely impact wellbeing, self-efficacy, and achievement. To combat these issues, a peer mentorship ‘buddy’ programme was piloted. This buddy programme formed buddy groups, consisting of one doctoral student (as the buddy group leader) with three to four pre-doctoral postgraduate students (as the buddies), which met fortnightly during the semester. Ten participants engaged in semi-structured interviews, and the transcripts were analysed using a thematic analysis approach. Identified benefits to participants of the buddy programme included improved social resources, expanded skillsets, improved confidence, increased enjoyment and motivation, career advancement, improved ability to navigate postgraduate applications, improved sense of belonging, improved coping through COVID-19 lockdowns, and increased collegiality. More specifically, benefits of the buddy programme, as voiced by the participants, included gains in wellbeing, academic attainment, and resilience. Given the estimated cost of NZD$400 per buddy group, as well as the openly provided resources developed from this study, tertiary institutions may consider implementing similar low-cost programmes to support their postgraduate student populations.

**Supplementary Information:**

The online version contains supplementary material available at 10.1007/s40670-024-02189-4.

## Introduction

Accumulating evidence indicates disproportionately high rates of psychological distress and mental ill-health among postgraduate students. Data acquired from postgraduate students in countries such as the USA [1], the UK [2], Australia [3, 4], and Belgium [5] point to a mental health crisis in graduate education. An absence of guidance and information, feelings of isolation, and the negotiation of multiple identities are among the challenges encountered by postgraduate students [6] that adversely affect student wellbeing [7]. The consequences of poor wellbeing among postgraduate students include limited academic productivity and attrition [8, 9]. There is a need for initiatives that enhance postgraduate student wellbeing and success.

Prior research identified several predictors of postgraduate student wellbeing, including the quality of relationships between students and supervisor(s) and faculty; involvement in activities; and concern for students as professionals [10]. Student wellbeing has been linked to improved student success, improved emotional intelligence, and self-efficacy [11]. More social support has been linked to higher wellbeing [12, 13], whereas a lack of social support is associated with lower wellbeing and higher prevalence of mental illness [14]. Social support is vital for postgraduate students; however, students may struggle with access to peer relationships [15]. Access to social support is particularly important for those at greater risk of social isolation, namely students belonging to minority groups, of lower socioeconomic or those who have international status [16, 17].

The postgraduate experience can be split into two divisions, pre-doctoral (encompassing Postgraduate Diploma, Honours, and Masters students) and doctoral (PhD students), to recognise the unique identities and challenges faced by these students. For pre-doctoral students, the transition to postgraduate study is erroneously assumed to be straightforward due to familiarity with the university environment compared to other educational transitions [18]. However, a multitude of complex factors negatively impact the transition to postgraduate study including social isolation, conflicting academic and personal responsibilities, and an absence of information provision [6]. Compared to pre-doctoral studies, doctoral study is typically of longer duration (at least 3 years), assumed to be more intellectually rigorous, and requires completion of a complex research project supervised by a faculty member. Additional challenges for doctoral students may include competition to find post-doctoral job opportunities, the low rate of pay, and difficult student-supervisor relations [19].

The development of programmes such as university promoted social support groups and peer mentorship schemes show promise to improve postgraduate student wellbeing [20, 21]. Improved self-efficacy, understanding of university processes, and sense of community are amongst the benefits conferred by such programmes [20, 22–26]. However, implementation of these programmes are hindered by large administrative load, operational costs, and time burden [15, 27]. Moreover, postgraduate student peer mentoring programmes focus largely on doctoral students despite considerable evidence indicating that pre-doctoral students need more support in the transition from undergraduate to postgraduate studies [18, 26, 28]. Additional limitations that may exist in postgraduate peer mentoring programmes include a lack of available time for meetings, differing expectations between mentors and mentees, and an absence of clear boundaries [20].

In the present study, we explored the retrospective views of students who engaged in a pilot peer mentoring scheme (referred to as the ‘buddy programme’). The programme aimed to provide the documented benefits of peer mentoring, while limiting administrative load, cost, and time requirements. Furthermore, all materials used in the programme would be available for use by institutions to implement their own mentorship scheme. This programme aimed to directly address problems facing both pre-doctoral and doctoral postgraduate students. Inclusion of pre-doctoral students in the programme may improve their transition to postgraduate study, and could provide them with an existing support network if they choose to continue to a doctoral degree. Doctoral students may enjoy the socialisation and sense of reward from helping others. We explored the experiences of participants in the buddy programme through semi-structured interviews to identity potential benefits and limitations of the programme for postgraduate students.

## Methods

### The Buddy Programme

Pre-doctoral students enrolled within the School of Medical Sciences at the University of Auckland and in their Honours, Postgraduate Diploma, or Masters degrees were invited to join the programme as pre-doctoral ‘buddies’ (mentees). The Honours degree programme combines 1 year of postgraduate-level coursework with a supervised research project. The Postgraduate Diploma typically involves 1 year of postgraduate-level coursework. The Masters degree typically involves 1 year postgraduate level coursework and 1 year of a supervised research project. Students may enrol in these programmes following completion of their undergraduate degree.

**Fig. 1:**
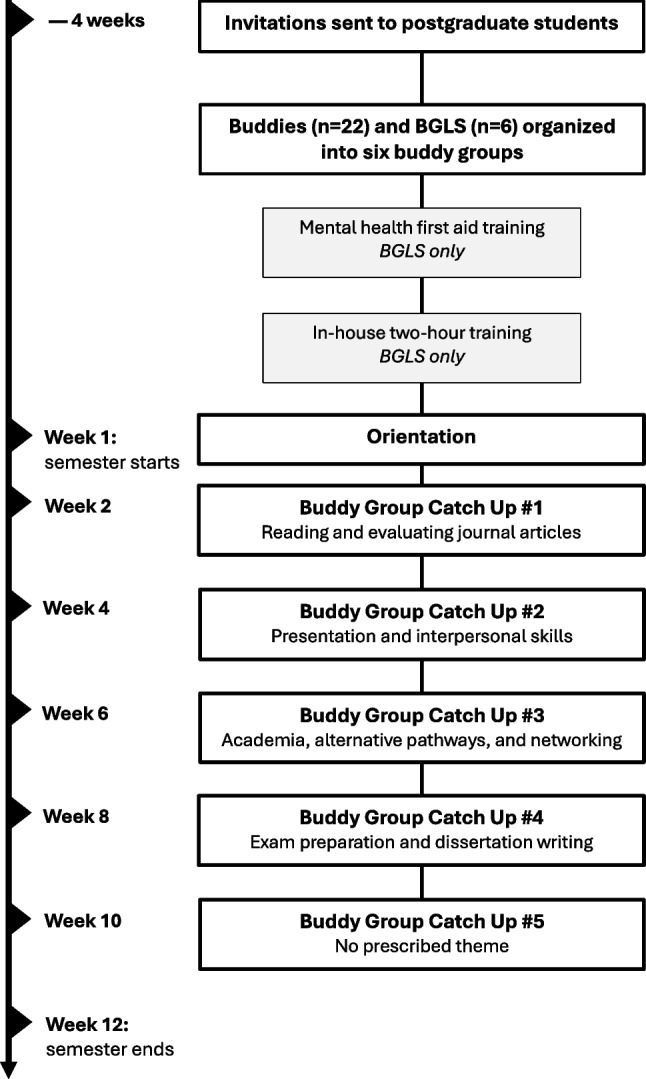
Structure of the buddy programme. BGLs (doctoral students) acted as mentors for the buddies (pre-doctoral students) through the 12-week semester. BGLS = buddy groups leaders (doctoral student mentors)

Doctoral degrees in New Zealand are typically 3- to 4-year degrees comprising research and typically no coursework. Doctoral students who had completed their provisional year (i.e. passed their 1-year confirmation review) were invited to join the programme as mentors to pre-doctoral student buddies and were designated ‘buddy group leaders’ (BGLs, affectionately pronounced *biggles*). MD and MD/PhD students were not included in the study. Invitations were sent 4 weeks prior to the start of semester. Eighty pre-doctoral students were invited to participate. Invitations for doctoral students were sent to a faculty-wide mailing list for postgraduate students. Twenty-six pre-doctoral students and 11 doctoral students applied to participate in the programme. Following closure of applications, the pre-doctoral and doctoral students were grouped based on their scheduling requirements and research interests (i.e. students with similar interests grouped together when possible). Students that did not respond to our emails, were ineligible to participate, or decided after their application not to participate were not included in the programme. The final six ‘buddy groups’ consisted of three to four buddies and one BGL each; a total of 22 included buddies and six BGLs. The BGLs were doctoral students in their second, third, or fourth year of study. The first event was an introductory orientation setting the expectations and boundaries of the programme. There were also two training sessions for the BGLs: a Mental Health First Aid Course [29] and an in-house two-hour training session run by the buddy programme coordinators. The structure of the buddy programme is shown in Fig. 1.

The buddy groups met fortnightly for a total of five ‘catch-up’ sessions across a 12-week semester with the following designated themes: (i) reading and evaluating journal articles; (ii) presentation and interpersonal skills; (iii) academia and alternative pathways, networking; (iv) exam preparation and dissertation writing, and no prescribed theme for the final catch-up. The session themes were decided following a meeting with the BGLs during their orientation session about challenges they faced during their transition to doctoral study. Catch-ups were held at the on-campus café with complimentary beverages or on video conference (due to COVID-19 restrictions). The buddy programme was implemented originally by PhD students (authors J.C. and J.P.) who facilitated three iterations (2021 Semester 2, 2022 Semester 1, 2022 Semester 2), and trained up a successor who facilitated it in 2023. The associated costs were NZD$250 per BGL (for the Mental Health First Aid Course), and roughly NZD$150 per buddy group ($6/beverage × 5 people × 5 catch-ups). The templates for emails, in-house training materials, and resources are found at this link: https://doi.org/10.17608/k6.auckland.21940016.v1.

### Participants

Following the end of the semester and the buddy programme, all participants were invited to participate in a semi-structured recorded interview. Four BGLs and six buddies were recruited and each were given a NZD$20 grocery store voucher after the interview. The transcript of the interview was sent back to the ten participants for validation. The research project was approved by the University of Auckland Human Participants Ethics Committee (UAHPEC22944).

### Research Questions and Domains

The core research questions aimed to ascertain participants’ perceived benefits of the buddy programme and how the pilot buddy programme could be improved. The semi-structured interviews comprised 23 questions across 9 sequential domains: (A) Context, (B) Expectations and Goals, (C) Value of Buddy Programme, (D) Motivation, (E) Self-Efficacy, (F) Achievement, (G) Leadership, (H) Wellbeing, and (I) Improvement.

The (A) Context domain was designed to understand the students’ academic background. The (B) Expectations and Goals domain aimed to identify the main desired benefits for the buddy programme. The (C) Value of Buddy Programme, (D) Motivation, (E) Self-Efficacy, (F) Achievement, (G) Leadership, and (H) Wellbeing domains intended to identify the most beneficial aspects of the buddy programme. Finally, the (I) Improvement domain was intended to capture problems and improvements of the buddy programme. Given the generic nature of the questions, both buddies and BGLs were asked the same semi-structured interview questions. These domains were chosen based on lived experiences of key informants of the postgraduate transition. We expected that participants in the buddy programme would likely experience positive impacts in these domains. To garner a broader, multicultural approach, we further consulted with independent student-facing advisers with expertise in student-centred research.

### Thematic Approach

The interviews were coded originally in NVivo (QSR, Doncaster, Australia) according to the nine a priori domains using a deductive framework within the prescribed hypotheses. However, many comments began to transcend the a priori domains. For example, the theme of improved social resources permeated throughout the a priori domains—students may have joined the buddy programme with the goal of widening their social circle, reported improved wellbeing from increased social support as a value of the buddy programme, as well as wanted more buddy groups as a suggestion of improvement. The initial coding allocation was developed to better describe the identified benefits of the buddy programme. The domains were morphed into a new framework via inductive thematic analysis [30].

## Results

The new inductively generated framework regarding the benefits of the buddy programme are included as follows (in the order of the most to least prominent theme):

1. Improved social resources
2. Expanded skillset and increased confidence
3. Increased enjoyment and motivation
4. Career advancement
5. Improved ability to navigate postgraduate applications and postgraduate life
6. Developed a sense of belonging
7. Improved coping through COVID-19 Lockdowns
8. Encouraged collegiality

Table 1 summarises each theme and the relevant findings and Supplementary Table 1 includes quotes as supporting evidence.

**Table 1:** BeBenefits of the Buddy Programme

Theme	Description
Improved social resources	Positive effects of improved social resources included feelings of being supported, improved self-compassion, reducing feelings of imposter syndrome, and stability from seeing a regular group of people
	The structured safe space of the buddy group catch-ups allowed participants to feel validated and to share academic and personal concerns they felt uncomfortable sharing with others
	The building of horizontal (between participants at a similar stage of study) and vertical (between participants at different stages of study) social relationships allowed for capability of seeding friendships
	Participants enjoyed making friends with both people in similar departments along with people in other areas whom they would not normally encounter—such that they could obtain better understanding of other peoples’ experiences
Expanded skillset and increased confidence	Improved communication skills such as active listening, leading the conversation, exhibiting empathy, reflection, and navigating social dynamics
	Increased confidence in academic skills such as thesis writing, using citation tools, figure creation, public speaking, organising articles, etc
	The improvement of such skills allowed BGLs and buddies to be able to see themselves in future leadership roles
Increased enjoyment and motivation	Enjoyment of the process of engaging in buddy group catch-ups led to improved motivation, better scheduling, or re-energised participants to continue their studies
Career advancement	To improve one’s *curriculum vitae* with evidence of service
	To explore or better understand one’s personal career trajectory
Improved ability to navigate postgraduate applications and postgraduate life	Participants reported improved clarity of the different postgraduate pathways afforded to students (e.g. Honours, PGDip, Masters, PhD) and their application processes
	Participants also reported more confidence and comfort in approaching relevant people (e.g. potential supervisors, other departmental staff)
Developed a sense of belonging	Participants reported a greater sense of belonging to a wider group, department, or community
Improved coping through COVID19 lockdowns	Improved motivation, social supports, and ability to continue their studies despite the COVID-19 lockdowns
Encouraged collegiality	The buddy programme was an outlet to encourage collegiality where participants were able to give back and to build towards better support systems for future students

*BGLS* buddy groups leaders (doctoral student mentors)

### Improved Social Resources

This theme was the most prominent and complex to arise from the interviews. Improved social resources manifested in four ways: (i) positive affect for individuals, (ii) a structured safe space to share concerns, (iii) the building of horizontal and vertical friendships, and (iv) offered perspective on individuals’ own experiences by comparing across different departments, degree structures, and disciplines.

#### Positive Affect for Individuals

Participants reported reduced feelings of imposter syndrome and improved self-compassion—e.g. treating setbacks with kindness and recognising one’s shared humanity [31]. An exemplar quote demonstrates this:

*“It made me feel more confident in the environment that I was in and therefore made me feel better about what I was doing and it made me feel like I could belong there, and I didn't have imposter syndrome as much anymore.”*

Further, participants reported that these social resources improved their wellbeing with feelings of being supported and the stability from seeing a regular group of people. An exemplar quote is shown as follows:

*‘I think the biggest thing was the social factor of it, just being able to have people to support each other. I didn't even realize that the two people that was part of [my] buddy group- they were in my classes and before that I thought I was all alone in those classes. Not many of my friends are doing the same thing as me so it was just reassuring to know that I had people around me.’*

#### Structured Safe Space to Share Concerns and to Receive Validation

The structured safe space at the buddy group catch-ups afforded participants more comfort in sharing their academic concerns in contrast to sharing with their supervisor—particularly if it concerned their supervisor. Aside from supervisory issues, participants also reported feeling more comfortable sharing emotionally difficult situations (e.g. embarrassing work situations, lack of motivation, and repeated experimental failures). This structured safe space allowed participants to better utilise their social resources to meet increased challenges and reduced adverse internal feelings. Furthermore, participants also felt these buddy catch-ups validated their concerns from shared hardships amongst other buddy group members. An exemplar quote is cited as follows:

*‘I think having that space to share your concerns and get that validation by people sharing their concerns back with you, it stimulates trust and a real openness and validates your experience, which I think in turn, increases your competence and even if it's not directly related to how I can undertake research I think feeling like you are capable of it, and by talking about it and talking about problems that you may have and potential solutions that you can come up with; I think puts you in good stead to feel more capable of problem solving.’*

#### Building of Horizontal and Vertical Friendships

The building of both horizontal friendships (i.e. peers at similar stages of study) and vertical friendships (e.g. BGLs at later stages of study) was particularly beneficial for participants. Buddies recognised the importance of having horizontal and vertical support networks as a protective factor to ameliorate encountered personal or academic problems. Buddies could also expand their social circles as their BGLs could introduce their buddies to mutual friends or departmental colleagues. Furthermore, these buddy group relationships often organically continued beyond the scope of the buddy programme. Participants felt able to continue contacting their buddy group members after the buddy programme had ended, felt more comfortable in their work environment due to these established relationships, and importantly, felt able to use these social friendships for support when faced with challenges. For example:

*‘just having someone who had already been through it that you could talk to, and outside of your lab as well; a few times I had issues that I didn't necessarily want to take my supervisors, because they were you know, maybe to do with my supervisors.’*

#### Offered Perspective on Individuals’ Own Experiences by Comparing Across Different Departments, Degree Structures, and Disciplines

Participants also felt it was beneficial in knowing the range of experiences across different departments, degree structures, and disciplines. In some buddy groups, participants belonged to the same degree structure (e.g. all buddies were Honours students completing research projects in similar departments) and in other buddy groups, participants belonged to varying degrees (e.g. across different departments or across different levels of study—e.g. masters versus honours). The advantages of groups with similar students meant that students could better relate to each other and understand each other’s experiences, and BGLs were able to tailor their help to all buddies more efficiently. Alternatively, the advantages of groups with dissimilar students were that participants could gain a wider perspective of how their own experience compared to others’ from different departments. As such, balancing the commonalities and differences between participants was often cited as an improvement, for example:

*‘Maybe doing more of a questionnaire beforehand and aligning the buddy group leader better with the student or the buddies that they have. So I know there was another girl in my group who was with the [another department], and so it wasn't very helpful for her- my buddy group leader couldn't really help her in the same way that she could help the rest of us so maybe having more of an idea of what students would fit best with an appropriate buddy group leader. I know I benefited a lot because my leader was in the same department as me.’*

Further suggestions for improvements are detailed in Supplementary Table 1.

### Expanded Skillset and Increased Confidence

BGLs reported improved leadership and communication skills. As the BGL, they practiced communication skills including active listening without judgement, leading the conversation by asking open questions, exhibiting empathy regarding problems facing their buddies, reflection skills, and navigating social dynamics within the group to ensure everyone was heard. An exemplar quote is shown as follows:

*‘it was really good – I did find myself listening more and more this time… I really tried to actively listen to the concerns of students and not insert myself into what they were experiencing- there's a fine line between sharing your experience to inform people but also not to discredit or devalue their experience to share with you, so I found myself putting more effort to actively listen and I really found that very easy to do by the end of our sessions.’*

Both BGLs and buddies reported feeling more confident in using academic skills such as writing one’s thesis, using citation tools, creating figures via software such as Biorender, public speaking, presentation skills, and organising readings. Though BGLs already had experience in these aspects, they often felt the need to revise their gathered resources in order to talk about these topics. This resulted in mini communities of enquiries within their buddy groups generating ideas for everyone to use. An exemplar quote is shown as follows:

*‘So definitely the citing tools that our buddy group leader introduced us to – that really helped me because I’ve got 100 references for my thesis so it was really helpful to just have a quick way to do that and an efficient way to do that. As well as getting to know those software tools like Biorender, for example, you can create your own diagrams, your own little cells; that's something that I'll be using in my introduction and I've already talked to my supervisors about getting a Biorender account or setting one up for the department.’*

Lastly, BGLs and buddies developed confidence in seeing themselves in a future leadership position. BGLs paralleled these buddy groups to starting their own lab groups; and buddies drew parallels to one day leading these buddy groups. This buddy programme evidently provided a strong platform whereby participants could build one another up to pursue future goals. For example:

*‘I would say that it helped my self-confidence, particularly around small group situations… I've been a part of several different lab groups and it's been interesting seeing how PIs go about managing their research teams and I've always wondered how I will be in that situation, so it was nice to think that this might be an early indicator, that it won't be a problem if I get into that situation, that I'd be able to do that as well, so yeah I would say it helped my self-confidence.’*

### Increased Enjoyment and Motivation

The buddy programme and its associated buddy catch-ups were enjoyable affairs which enhanced intrinsic motivation in participants’ own studies. All participants felt that the time commitment of the buddy programme meant that they had to better manage their time and schedule around these catch-ups, and forcibly take a break around lunchtime. None of the participants reported feeling the buddy programme detracted from their studies. BGLs reported that the affirmation received from knowing they had positively affected their buddies gave them a sense of achievement and re-energised them in their work. For example:

*‘one thing that was quite nice- the affirmation that I got from [buddies], that I was doing a good job as their BGL. Because that was my goal to make it a nice environment for everyone to have a little bit of downtime and share problems or ask questions if they had any.’*

### Career Advancement

Career advancement was cited as a benefit for both BGLs to improve their *curriculum vitae* with evidence of service as well as for buddies to explore or better understand their personal career trajectory. The buddy programme facilitated interactions between pre-doctoral students and doctoral students as well as hosted career seminars aiming to explain the various ‘40–40-20’ research, teaching, and service aspects of the academic career. Therefore, these buddy programme activities afforded buddies much better clarity in what they wanted for themselves. Further, some participants remarked that the buddy programme could be improved with more resources (e.g. workshops, seminars) on other non-academic research careers. An exemplar quote below summarises the perspective of buddies with regard to career advancement in this buddy programme:

*‘Now I know a lot more about what a career in research actually means and what that looks like, as well as in academia, as well as the fact that different institutes and universities have different rules and every place is different, different countries as well, so yeah I got to know what postgraduate life really means and even after postgraduate life what that means, like how to get a job and all that stuff.’*

### Improved Ability to Navigate Postgraduate Applications and Postgraduate Life

Participants reported improved clarity of the different postgraduate pathways afforded to students (e.g. Honours, Postgraduate Diploma, Masters, and PhD) as well as their application processes. For example, participants were better informed about the grades they needed to achieve to apply for certain programmes, timelines and deadlines for scholarship applications, etc. Furthermore, participants also reported more confidence in contacting lecturers and reaching out to other members of their department. These benefits extended to BGLs, as they were careful to give updated and correct information. An exemplar quote is shown as follows:

*‘I think being in a position where you are a leader and you need to inform people about research in the academic space, you need to be well informed that you're not giving students misinformation so I tried really hard to educate myself on aspects of university life that I wasn't as familiar with and that also included career options for people in research. Obviously, I can only speak to things that I know, but I also tried to provide information on what else is out there, based on my experience but also experience of my peers and my friends and what they have done just to give a more well-rounded answer and information to students so, yes, [the buddy programme] really helped me navigate postgraduate space by educating myself on all aspects of postgrad space.’*

### Developed a Sense of Belonging

Participants reported feeling a greater sense of belonging to a wider group, department, or community through the buddy programme. BGLs enjoyed the camaraderie amongst other BGLs and how they could support each other along with supporting their respective buddies more. Buddies also enjoyed feeling more connected with their Faculty such that they could feel more comfortable in their space. For example:

*‘I was looking to become more involved in the Faculty of Medical and Health Sciences, I have friends that are also doing honours with me, but I did feel a little bit more isolated this year coming into the lab and sort of felt very disjointed compared to previous years, so I wanted to have a little bit more of a support system, a little bit more of community feel within the Faculty.’*

### Improved Coping Through COVID-19 Lockdown

Participants reported an improved ability to cope with studying specifically throughout the COVID-19 restrictions in New Zealand. The lockdown in August 2021 in New Zealand forced the remaining buddy catch-ups to continue solely on Zoom. Incidentally, participants reported that these catch-ups encouraged buddies and BGLs to talk with people outside their immediate social circles (i.e. family, flatmates, and close colleagues) and the social interaction helped motivate participants. For example:

*‘I think the whole lockdown situation is quite difficult to get in touch with people and I think it can make you feel a little bit isolated. And so to have people there that you know you can contact for something or to show you who you should be getting in touch with or whatever has been really good throughout lockdown.’*

### Encouraged Collegiality

The buddy programme encouraged collegiality amongst postgraduate students where participants could give back and build towards better support systems for future students. For buddies, it was encouraging that there were so many BGLs willing to volunteer to help their colleagues. For BGLs, there were three main reasons underlying why BGLs joined the programme: a desire to have better support systems within the University, having encountered difficulty in their studies and wanting to mitigate the likelihood of negative experiences for future students, and also giving back to future students because they had received support from others. The buddy programme gave BGLs an outlet for contributing to a collegial environment. For example:

*‘it’s also a good way to give back to undergrad students as well and people like me have had the privilege of going through this academic pathway. And I’ve done so well, partly due to my own efforts, but also because I’ve had a lot of people around me to really help me through that journey and so it’s definitely an awesome way to give back into it and ensure that you're that person for someone else.’*

## Discussion

The key ideas emerging from the findings can be summarised according to the value of the programme and the potential improvements to the programme. Lastly, we will consider the limitations of the present study.

### The Value of the Buddy Programme

The identified benefits of the buddy programme align with the literature of improved student wellbeing following participation in a peer mentorship group [21]. The major social supports provided to participants of this buddy programme directly increased the social resources of students affording them greater capacity to meet challenges [32]. Prior work indicates that social support also has a buffering effect on stress among first year graduate students, such that social support improves satisfaction with graduate school even when highly stressed [33]. Though the buddy programme did not decrease demands on students, the increase in social support is expected to increase their ability to cope with these demands [33]. Social support may also increase academic engagement and prevent emotional exhaustion, a precursor to burnout [34]. In addition, the buddy programme appeared to increase enjoyment and motivation—key factors for academic success and wellbeing [35]. The incidental finding that the buddy programme was able to improve coping through COVID-19 restrictions is also notable. Given the documentation of the difficulty in studying throughout the COVID-19 pandemic [36–38], this would suggest that the buddy programme could act as a protective factor for students’ wellbeing during times of chronic stress.

Both BGLs and buddies reported feeling increased self-confidence. Greater self-confidence is of importance given the well-documented effect of imposter syndrome on postgraduate students [39]. Imposter syndrome is a pattern of thinking common amongst postgraduate students characterised by feelings of intellectual inferiority and academic-unpreparedness [40, 41]. Indeed, two of the participants noted the benefit of the buddy programme with specific reference to imposter syndrome. The expanded knowledge and skillset affected both buddies and BGLs giving them increased confidence in their communication, leadership, and academic skills. The improved academic skills directly address the importance of writing skills for graduate student wellbeing [14]. Collectively, the expanded knowledge and skillset produced feelings of an academic identity which is well-known to be both a strong predictor for student success [14] and associated with graduate student achievement [42].

It was also identified that a strained supervisor-student relationship could be difficult to navigate, and the provision of an intermediary support person could greatly benefit students—rather than directly going to senior academic staff such as the head of department. Help-seeking behaviour is low among graduate students [19]; the provision of formalised peer support could be a useful mechanism to increase help-seeking. Lack of awareness of services available is a reason for low help-seeking among university students [43]. The transfer of knowledge from BGLs to buddies likely increases awareness and a sense of being supported. The creation of a safe space where feelings can be discussed openly may also reduce the stigma associated with mental health difficulties known to reduce help-seeking in university populations [44]. The structured safe space of the buddy group catch-ups also allowed participants to better practice self-compassion. The realisation for buddies that their colleagues also suffered setbacks in their studies was important in recognising one’s shared humanity and treating oneself with kindness—as entailed in self-compassion [31]. Our interview observations suggested that the participants were able to better practice self-compassion, which may improve self-esteem and wellbeing [45]. Furthermore, the creation of a safe space is contrary to the harmful competitive environment often experienced by graduate students [46]. The competitive environment, including fears of admitting to problems or feelings of inadequacy, is likely among the reasons for doctoral student attrition [46].

While previous work suggests time is a significant barrier to the success of peer mentorship schemes [27], none of the participants in the current study identified time as an obstacle. Rather, participants identified the time required by the buddy programme as conducive to their wellbeing through mandating a short break and also re-energised them for work. Therefore, the frequency and length of meetings required by the buddy programme may be considered a strength of this programme. Furthermore, the buddy programme is facilitated by student peers. These peers are volunteers; there is no payment nor use of faculty time required to facilitate the programme.

### Potential Improvements to the Buddy Programme

The most prominent improvement suggested was expansion of the programme and reaching more people. However, it must be noted that postgraduate students are a more heterogeneous cohort compared to undergraduate students with students studying widely varying research topics, research group environments, and varying enrolment situations (e.g. full-time versus part-time) [47]. Successful peer mentoring schemes are moderated by factors such as ability to commit time, closeness in age, and similar programme of study [48]. Given that the success of peer mentoring schemes is dependent on the ability to commit time, it is not advisable for the buddy programme to become mandatorily prescribed. As such, expanding the participation of the buddy programme lies at the intersection of faculty staff support, provision, and recommendation as well as student eagerness. Furthermore, given that our results found some participants feeling less supported due to being in a different department, we suggest that these programmes are not run too broadly across different faculties where commonalities in experiences are sparser.

Asynchronous avenues for buddy programme participants to connect is another operational improvement to the buddy programme. Creating a platform for chat functionality (e.g. via Discord™, Teams, Slack) would be a low-cost method to allow buddies and BGLs to communicate with each other. Discussion boards may enhance a sense of belonging and develop community for buddies. For BGLs (i.e. doctoral students), past research has identified the need for developing scholarly communities as learning environments for the benefit of doctoral students [49]. In the absence of other institutional communities that may fill the need for a scholarly community, this asynchronous platform and buddy programme community could potentially fill the scholarly community that both respects the seniority of doctoral students in the buddy programme but also provides them connection to other peers (i.e. BGLs) with similar experiences.

### Limitations of the Present Study

The present study lacked a control, therefore, it is difficult to determine the magnitude of impact of this buddy programme. Furthermore, we acknowledge that a small proportion of students were included in this study (6/22 buddies and 4/6 BGLs). Also, the present study was conducted during the acute stages of COVID-19 lockdowns in New Zealand and thus may present a unique circumstance that is not easily replicated. Nonetheless, these ten participants provided in-depth qualitative information that would not be readily available using other research methods, such as surveying the wider population. Furthermore, the present study demonstrates a student-led initiative totally reliant on volunteerism, though the resources developed are intended for translation into institutional programmes with more stable funding mechanisms.

## Conclusion

The present study demonstrates the feasibility and value of a peer mentorship programme for postgraduate students and provides template resources. The cost of this buddy programme was approximately NZD$400 per buddy group. The benefits included improved social resources, expanded skillsets, increased confidence, increased enjoyment, increased motivation, career advancement, improved ability to navigate postgraduate processes, a sense of belonging, increased coping through COVID-19 restrictions, and increased collegiality. These benefits, though largely financially intangible, provided value that likely translated into improved student wellbeing. Graduate students are vulnerable to mental health concerns and social isolation, to the extent that academics cite a ‘crisis’ in graduate education. Thus, it is vital to implement programmes that improve support, coping, and postgraduate student wellbeing. The current study demonstrates a peer mentorship programme suitable for postgraduate students that successfully improves social support, self-confidence, and wellbeing. Given the responses of participants, we expect the programme may also aid in achievement and reduced attrition though further investigation is needed to confirm this. Universities may consider implementing similar programmes to support their postgraduate students.

## Supplementary Information

Below is the link to the electronic supplementary material.

Supplementary file1 (DOCX 44 KB)

## Data Availability

All data generated or analysed during this study are included in this published article and its supplementary information file.

## Declaration

The materials used to run this buddy programme are freely available at this doi link: 10.17608/k6.auckland.21940016.v1.
