# Supplementary material for: The Value of a Peer Mentorship Programme for Postgraduate Students in New Zealand: A Qualitative Study
Source: Med Sci Educ. 2024 Oct 12;35(1):319–29. doi: 10.1007/s40670-024-02189-4 (PMC11933583; doi:10.1007/s40670-024-02189-4)
Supplement: Supplementary file 1 — Supplementary file1 (DOCX 44 KB) [file 40670_2024_2189_MOESM1_ESM.docx]

# Table S1: Benefits of the Buddy Programme With Quotes

| Theme Description | Examples |
| --- | --- |
| Improved social resources Participants reported improved social resources which had positive affect on themselves, for example, feelings of being supported, improved self-compassion, reducing feelings of imposter syndrome, and stability from seeing a regular group of people.  The structured safe space of the buddy group catch-ups allowed participants to feel validated and to share academic and personal concerns they felt uncomfortable sharing with others.  The building of horizontal (between participants at a similar stage of study) and vertical (between participants at different stages of study) social relationships allowed for capability of seeding friendships.  Participants enjoyed making friends with both people in similar departments along with people in other areas whom they would not normally encounter – such that they could obtain better understanding of other peoples’ experiences. | - 1. “I think having that space to share your concerns and get that validation by people sharing their concerns back with you, it stimulates trust and a real openness and validates your experience, which I think in turn, increases your competence and even if it's not directly related to how I can undertake research I think feeling like you are capable of it, and by talking about it and talking about problems that you may have and potential solutions that you can come up with; I think puts you in good stead to feel more capable of problem solving.”   2. “it's kind of nice to know that other people are in the same boat, and so, when we’d have our buddy meetings, it was nice to hear that I wasn't the only one struggling to find a bit of motivation in the middle of lockdown but something we did as a buddy group was we organized some- I think we called them writing workshops. Just zoom meetings where we all had a goal that we needed to get done for the next hour. That was actually really, really helpful and we would always choose a time in the day that we struggled with sort of motivation, or whatever, to make sure we actually got stuff done then.”   3. “I really value doing programs like this because I think it really humanizes the experience of research and academia in general. I think everyone has something to learn from other people also taking this journey of research and it's easy to feel like you're the only one going through it in this particular way. Everyone [has] varying degrees of imposter syndrome, so I think having a dialogue with people who are at various stages of their career is always super helpful because it makes the process less scary because you know that everyone is experiencing similar things.”   4. “It made me feel more confident in the environment that I was in and therefore made me feel better about what I was doing and it made me feel like I could belong there, and I didn't have imposter syndrome as much anymore.”   5. “So obviously COVID has been strange right and we did half of our buddy stuff online so that was just nice catching up with other people. Obviously, it was a struggle with my research put on hold fully with the whole lockdown thing; my samples had to get thrown out and we had to order new ones- there's a lot that happened, so sharing that with people other than my supervisors was nice, being able to talk about it, being able to like rant about it, and other people who are on the same boat as well. One of the other girls, who is also doing an honours year; her research will also put on hold because of COVID. So it was just things we talked about, laughed about, we of course talked about so many unrelated things to our research, it was just a nice program where we all motivated each other and we're also just sharing our experiences honestly… in a not academic way. Because, obviously I can't say that to my supervisors, but I can with another group of students.”   6. “I did have a bit of a struggle with presenting a paper and my professor at the time, [they] were just asking all these questions, I was getting insanely nervous and I couldn't answer anything. So that was something that put me down, and that was at the beginning of my honours year, so I remember talking to the buddy group about it, telling them this happened, this happened; it really put me down, and then they were also just sharing their own experiences and actually our buddy group leader, she said that she was giving a presentation at the beginning of her PhD and she fainted in the auditorium and they had to get her out so it was a funny story but it just made me feel like, oh everyone goes through these like hiccups during their journey or their studies, so I think that just helped me realize like oh it's not too bad if I didn't do too well at first”   7. “Knowing that other people were struggling similar to me, it felt like I wasn’t such a failure. So it made me feel like I can stick it out since other people were also in the same situation.”   8. “just having someone who had already been through it that you could talk to, and outside of your lab as well; a few times I had issues that I didn't necessarily want to take my supervisors, because they were you know, maybe to do with my supervisors.”   9. “But also, I just liked that it was quite reassuring, knowing that I had other people who were feeling the same way, or just knowing that I could talk to them or learn a few things – being able to learn from them and knowing that some things that I was doing that could maybe help them as well, so I think that was helpful. It definitely helped me learn a few tips to improve my studies.”   10. “I still run into some of my buddies every once in a while, and I appreciate how all of the BGLs, we would get together and have our own debriefs and that was very friendly so I’ve definitely made a few friends out of doing the buddy program so that’s been nice.”   11. “We had a chat with the other buddy group leaders who I've become good friends with and getting their perspectives or takes on how they are navigating the postgraduate space and also the buddies are welcome to reach out to the buddy group leaders anytime they're having an issue.”   12. “I think it's nice having more familiar faces around and at the start of the Semester, when we were still on campus that was really nice, because the people that were in my group, I didn't actually know before the program so yeah, having a few more familiar faces is really nice.”   13. “It improved my sense of social support and seeing that there were so many buddy group leaders, they obviously had to volunteer because they wanted to help people. It made me feel more comfortable, knowing that there are people that you know they're just happy to answer your questions and help you out.”   14. “I was going into honours I didn't realize how many of my experiment we're gonna go awfully wrong and… [my BGL] again had been through it, so it was just nice to hear that it's not me that's cursed. It's just science.”   15. “I think at the beginning of the year I was definitely questioning: I don't know if I can study anymore- this is really rough and now I'm a bit more motivated to keep going just because, like I’ve learnt a lot from just knowing that I'm not alone   16. “I think the biggest thing was the social factor of it, just being able to have people to support each other. I didn't even realize that the two people that was part of [my] buddy group- they were in my classes and before that I thought I was all alone in those classes. Not many of my friends are doing the same thing as me so it was just reassuring to know that I had people around me.”   17. “I made some new friends and I can contact my mentor whenever I need help in that space, like, I know that I have someone who has been there, that that I can reach out to at any point so yeah.”   18. “I thought it was a great way to meet people that were doing things that were different to what you were doing; meet people who were doing their PhD and like, my buddy group leader knew a lot of people that I was working with or sitting with in the office, so that was quite nice to be able to meet people through them or just feel more comfortable in that environment.”   19. “this buddy program was just something different. I got to learn about other people's projects, other departments, other students, other people's experiences. So I got an overview of what the postgraduate experience should be like instead of just my own experience, so yeah, it was nice to see things from other people's perspective and experiences, rather than just my own, and that was nice because we were four different people in this group.”   20. “…just having someone who had already been through it that you could talk to, and outside of your lab as well, like a few times I had issues that I didn’t necessarily want to take to my supervisors, because they were maybe to do with my supervisors”   21. “I wasn't really sure where I wanted to go next and so I think it was mainly hoping to get a little bit of insight and just be able to talk to someone later in their research career and to see if that was something that I could be interested in the future. I guess [I was] hoping that there would be someone to give me a little bit of guidance.”   22. “I'm in the buddy program [because] I wanted to be involved in something else, postgraduate-like. To interact with other people who are doing something similar, and the fact that the buddy group leader was a PhD student that would give me some insights into what a PhD would look like or their experiences. And also it seemed really nice to get to know people who are on the same boat as me as well as learn new things, and it also looked like it was one of those things where it wasn't a huge commitment, it was every two weeks we'd meet for an hour, we met online during the whole lockdown so it was something I could keep up with. It wasn't something I had to always go always because that can be really difficult - that's sometimes why I don't join a lot of things because they're always spamming you with emails always doing things. So it was nice that this was something that happened every few weeks and we would catch up on, I also wanted to be part of the buddy programme just because it seemed like a good idea, just the way it was presented - everyone was really nice. And yeah it just seemed like an inviting thing to do.”   23. “I was not really sure with what I’m really getting myself into for postgrad. I know what's written online and what the lecturers and people have to say, but I was interested in hearing first-hand experiences from people who have been doing postgrad and people who are doing a PhD and stuff like that, to see what the next step after PGDip and Masters would be like.”   24. “I was hoping to hear the experiences from postgrad students, people who have done their PGDip, or masters or honours or PhD currently and I was hoping to also meet other postgrad students who are doing the same degree or you know, in the similar sort of stage as me to see how they're finding it and maybe just meet new people in that sort of area.”   25. “It was nice being able to see a regular group of people that you could chat with. Because online lecturing this semester; you don't get to see everyone and it was nice to be able to keep in contact with people and touch base”   **Improvements**   - 1. “yeah maybe bigger groups as well, might be helpful because I only had three buddies and that was a suitable but I think having a larger, more diverse group would really help so they can engage with each other but also different points of view, which I think always results in richer dialogue and experience”   2. “I happened to have all honours students in my group so there was some other feedback we talked about whether it would be better to mix up the degrees or keep them separate, so we can do more tailored help.”   3. “Maybe doing more of a questionnaire beforehand and aligning the buddy group leader better with the student or the buddies that they have. So I know there was another girl in my group who was with the [another department], and so it wasn't very helpful for her- my buddy group leader couldn't really help her in the same way that she could help the rest of us so maybe having more of an idea of what students would fit best with an appropriate buddy group leader. I know I benefited a lot, because my leader was in the same department as me.”   4. “I think there's always opportunity to stratify even further like having students [from] the same background grouped together. Obviously, you don't want to segregate people, you want to expose them to diverse opportunity but in saying that people from the same background will have more overlap in their experience as well, so I think the bigger the program gets I think there'll be more opportunity for that collaboration but we'll see.”   5. “One of the discussions I had with the other buddy group leaders was around … in the sub doctoral space there's postgrad diplomas, there are master students and honours students and all these students have different needs, different goals, different requirements, and one of the things that… I felt during my buddy group was that I couldn't tailor my responses or my knowledge to these specific categories of sub doctoral students because there was a mixture in my group. I couldn't tailor my knowledge to them and the feedback that I got from them was that it didn't feel useful for them, because it wasn't tailored to them.”   6. “I was thinking it might be good to have your buddy group leader be doing a similar sort of thing that you would like to be doing in the future, but at the same time, there are so many aspects that carry over whatever specialty that you study or research and the same goes for the people in the buddy group, but I think it's good that I got to meet a variety of people so I'm not sure if that's an improvement. So maybe it’s better to keep it that way. I’m not sure.” |
| Expanded Skillset and Increased Confidence Improved communication skills such as active listening, leading the conversation, exhibiting empathy, reflection, and navigating social dynamics  Increased confidence in academic skills such as thesis writing, using citation tools, figure creation, public speaking, organizing articles, etc.  The improvement of such skills allowed BGLs and buddies to be able to see themselves in future leadership roles. | - 1. “Communication and active listening, engagement - those are all skills that can be sharpened time and time again. And this was just one avenue, in which I could do so.”   2. “it was really good – I did find myself listening more and more this time… I really tried to actively listen to the concerns of students and not insert myself into what they were experiencing- there's a fine line between sharing your experience to inform people but also not to discredit or devalue their experience to share with you, so I found myself putting more effort to actively listen and I really found that very easy to do by the end of our sessions.”   3. [how did the buddy program impact your confidence and ability to lead a group?] “Definitely positively so, especially the first few sessions, I was more leading the conversation trying to ask open questions and get the conversation rolling and just the fact that it got easier, I think, is further growth in my confidence as well. So yeah, definitely good.”   4. “in terms of my personal development, I think I built up my self-confidence, my listening skills, my reflecting skills and just my overall facilitation and support skills- which I will be able to apply in my postgrad studies or just outside when I'm navigating relationships with other people.”   5. “So definitely the citing tools that our buddy group leader introduced us to – that really helped me because I’ve got 100 references for my thesis so it was really helpful to just have a quick way to do that and an efficient way to do that. As well as getting to know those software tools like bio render, for example, you can create your own diagrams, your own little cells; that's something that I'll be using in my introduction and I've already talked to my supervisors about getting a biorender account or setting one up for the department.”   6. “in terms of some of those software skills, some of those things to help with writing and also, we got a few documents sent to us of how to write a thesis or how to search up articles on the library services at uni and although I already knew this stuff, I didn't really know how to apply or how to use it. So it was really helpful that [our BGL] showed us how to do that”   7. “[Our BGL] gave us helpful tips in terms of writing and how to go about gathering information for writing, like organizing the readings that you've done and sorting the articles and stuff like that and getting the articles that have come out and knowing where to find those.”   8. “[Our BGL] was really, really helpful on working out how to structure presentations and sorting out that kind of thing and practising with her and speaking. I'm not very good at public speaking so…”   9. “Yeah public speaking and learning how to like present your work to your lab group and to larger groups. I think it was good to get practice with the presentation… public speaking is one of my least favourite things and so to do that and practise for future because I’m sure I’m gonna have to present my work, the actual things in future, so to have the practice.”   10. “I guess it was even just down to like tips on presentations which I'm still using now, how to retain or better understand a research paper when I'm reading it and how to tackle that, picking out relevant information, as well as just like, planning… I think one of the last sessions was bringing things together for exams and dissertations if I had to do one and how to schedule things”   11. “With research we did get really helpful advice on how to find papers and how to connect papers to other papers, if that makes sense- so you're forming a network of information.”   12. “The part that was most helpful to me was definitely around HealtheX [a Faculty-wide student conference for students to practice their presentation skills]. So I did a presentation this year, and my buddy group leader was really, really helpful helping to prepare us for that and, doing extra sessions, so that we could prepare with her and then another girl for my group ended up winning it so that was quite nice. But we like all got to practise together.”   13. “We were given certain topics to talk about so I used to do a little bit of research around the topic just to hold that discussion together. So I guess I think it's sort of impacted on what I did for my own academic work, because I was able to use that knowledge for myself as well… I certainly think that because we were given topics to discuss and you get knowledge around these topics, so I was able to use that knowledge, so it did drive me to succeed and aim higher and achieve higher.”   14. “it was good for me to see different things that students struggle with so that if I had the power to look after a teaching course, for example, I could potentially I would know more of what the students’ struggles might be and address them prior to them becoming struggles. But I admit that I think that that would be a terribly hard thing to do, because there's give and take with everything that you do in any course, so I think with every student that you might help there'll probably be another who struggles in a different way. But I think just in general understanding the struggles of students in those courses and in those situations is an important thing for all academics, so that they can be more better navigated I suppose.”   15. “I would say that it helped my self-confidence, particularly around small group situations… I've been a part of several different lab groups and it's been interesting seeing how PIs go about managing their research teams and I've always wondered how I will be in that situation, so it was nice to think that this might be an early indicator, that it won't be a problem if I get into that situation, that I'd be able to do that as well, so yeah I would say it helped my self-confidence.”   16. “I think before I kind of [thought] I'd be more of a member than a leader, but now because I'm a bit more confident in myself and like where I'm going that I feel like I can help people and I think it just helped with the confidence and now I'm less afraid of asserting myself. I think that's kind of helped – still a work in progress, but it was definitely a start.”   17. “Probably not like teaching me leadership skills but like if I was in the position where I had this offered to me to be a buddy group leader, I think, from what I've learned in the course like I definitely want to sort of go and like be that leader for the buddy group.”   18. “[The buddy programme] did give me more of an idea of what it was like to do a PhD and how involved I would be with leading or teaching during my PhD. But that did make me think about more leadership roles that I could be doing in the future. One day, I would like to be a buddy group leader is one example I guess.”   **Improvements**   - 1. “I think the benefits of the program would have been better for the students if that was in semester one, but I understand they were concerned about the higher workload for the students in semester one. But I just think a lot of the skills to teach them they already had to figure out by themselves. But yeah I think in terms of my time and my stress level- no, it was fine, it was almost a stress relief sometimes to be able to talk to someone” |
| Increased Enjoyment and Motivation Enjoyment of the process of engaging in buddy group catch-ups led to improved motivation, better scheduling, or re-energised participants to continue their studies. | - 1. “one thing that was quite nice- the affirmation that I got from [buddies], that I was doing a good job as their BGL. Because that was my goal to make it a nice environment for everyone to have a little bit of downtime and share problems or ask questions if they had any.”   2. “It's certainly enhanced my postgrad life- I didn't really find it a burden on my time doing these sessions, so I think I certainly had a very positive experience and I enjoyed those sessions- our discussions with my buddies.”   3. “I find that just makes for a nice break in my day so the fact that we can schedule it over lunchtime made it very easy. It kind of forced me to have lunch which is probably a good thing. So no I don't feel like it took away from my PhD time.”   4. “I think seeing the reciprocation of the buddies was really encouraging to know that they were valuing these sessions, as much as I was, and they kept coming back to each session that we had timetabled was really good, so I never felt that I was putting in more effort than they were - it really felt mutual. I think we definitely built a good relationship like that and so I knew that I was capable of doing the job well and to get that response was really validating and it definitely contributed to self-confidence.”   5. “We had a couple of meetings before lockdown and we had a few after lockdown so I could see a huge difference in my motivation to do my coursework and my research. So when I used to have these meetings, motivation was a big discussion with the buddies that I was [the] group leader of. They came up with some really fantastic ideas on how to get yourself motivated during lockdown and how to pursue your coursework, so I think yes definitely, [it] did influence and sort of energized my research and how I could set deadlines for myself.”   6. “I found it quite energizing because it happened to be in the majority of it in lock down. Just having something scheduled on your calendar really helped me plan my day around it. And yeah, again like I feel like I had a little bit of pressure, but in a good way to succeed in my goals, so I can be a better mentor for them as well.”   7. “It was only one hour every two weeks, so I didn't feel that impacted or took away from study time at all. I would just plan- if I was in the lab, I would plan my experiments around going to the group meeting. And of course we got to pick when our buddy group time was so it's not like I had anything conflicting with it”   8. “This whole postgraduate year has definitely been a really nice experience that I’ve enjoyed; the buddy program was nice that we got to speak to people, we got to learn things; I definitely enjoyed all the meetups that we had- it was just really nice. It was surprising that [it] was only an hour [and that it] went by so quickly. So I definitely say it was nice because it just added something different to my usual routine in my lab and with my normal day to day life but that's how I enjoyed it.”   9. “I really wanted to foster an environment with these buddies that allowed us to [talk about their problems]” |
| Career Advancement To improve one’s *curriculum vitae* with evidence of service.  To explore or better understand one’s personal career trajectory. | - 1. “it’s also just good practice to upskill yourself and add to your CV”   2. “and also, I guess, I don't know if it sounds bad but the CV and academic service is always important.”   3. “So I didn’t really have any expectations, because I had no idea what exactly this was but I think the buddy program was definitely more of a social thing but also [an] academic thing as well, like you learn a lot through it. So my only expectation I [had] was just to know a bit more about what a career in academia means or what a career in research means. As well as just gain those insights from someone who's already doing that, so that was my only expectation and goal, and I think that did happen – I got to know a lot more than if I were to just go online and look up what I needed to know.”   4. “I really found a lot of information, new information that I had no clue about the PhD, Postdoc, Academia/lecturer pathway. I really had no clue what that entailed because at the moment I’m thinking I would like to be continuing in academia and there's a lot of jobs out there, that people can tell you what they do in their jobs etc. but there wasn't really that much information for the academia side of things, especially in scientific biomedical research, so I found that really helpful to see a broad scope of the things that I would be getting involved in if I did decide to pursue academia further so I found that really helpful”   5. “I was feeling a lot of uncertainty about where I was going, especially since like postgrad is quite a bit different to undergrad so I guess to have a little bit more certainty where I was going or if I was on the right track. I guess expectations for myself were to make some friends and maybe learn a few things that I weren't aware of before but also learning from [other people] basically”   6. “in academia like, how do you become a lecture, how do you become a professor, how do you become an associate professor- all those things you don't really know that you don't know. It was just good to know you need to publish this amount of papers, you need to have a score of this, you have this many students; so it was really helpful because I didn't know it takes a while to get into that position, but I had no idea what sort of things you had to go through to be qualified for a position like that. So that was really helpful; a PhD is obviously not enough- there's a lot that you need to do to get into academia. So that was for me really useful and it was just a good program overall- each time we met every session they just talked about something briefly and but in detail.”   7. “Now I know a lot more about what a career in research actually means and what that looks like, as well as in academia, as well as the fact that different institutes and universities have different rules and every place is different, different countries as well, so yeah I got to know what postgraduate life really means and even after postgraduate life what that means, like how to get a job and all that stuff.”   **Improvements**   - 1. “I think the only thing I can think of is to have more information on other possible careers… I probably thought there might be other options that may have an element of research, but not necessarily at the university.”   2. “I would have wanted a bit more information or I would have wanted maybe a more extensive… I felt like it was a nice program; it was really, really fun, really informative, but maybe more could have been just put into it – just in terms of detail or maybe workshops, seminars” |
| Improved Ability to Navigate Postgraduate Applications and Postgraduate Life Participants reported improved clarity of the different postgraduate pathways afforded to students (e.g. Honours, PGDip, Masters, PhD) and their application processes.  Participants also reported more confidence and comfort in approaching relevant people (e.g. potential supervisors, other departmental staff, etc.) | - 1. “I think being in a position where you are a leader and you need to inform people about research in the academic space, you need to be well informed that you're not giving students misinformation so I tried really hard to educate myself on aspects of university life that I wasn't as familiar with and that also included career options for people in research. Obviously, I can only speak to things that I know, but I also tried to provide information on what else is out there, based on my experience but also experience of my peers and my friends and what they have done just to give a more well-rounded answer and information to students so, yes, [the buddy programme] really helped me navigate postgraduate space by educating myself on all aspects of postgrad space.”   2. “[The buddies] had some questions about like applications for future studies which I had to research and try and find them the right advice. I think yeah, [the buddy programme] definitely taught me a bit more about that process and timelines and deadlines, and how to apply for scholarships and things like that.”   3. “there are certain grades that you need to get to get [into] research and all of that stuff through the university. I think some of that stuff I didn't know completely and so having someone that had been through that process and saying okay well after this stage, you need these sort of grades, and these are the options if you don't get those grades. At the same time, I was thinking I don't know if this is something I really want to go into or not. Like sort of having that in the back of my head, knowing that as a backup option or something.”   4. “I would say I already had a pretty clear view of what I wanted to do in the future, before I came to the buddy program. But talking to someone who was already doing a PhD did give me a lot clearer view of what my lifestyle would be like and sort of helped prepare me for more of a realistic view of what I was going to be entering into. My buddy group leader was also really interesting because I got to learn about how she ended up in the PhD program and she did like a Masters to PhD transition, or converted her masters to a PhD, which was something I'd never heard before so that was really interesting to know and sort of gave me another pathway to think about.”   5. “I think especially in undergrad, postgrad seemed like this huge thing, super serious so I was a little bit wary coming into it, so I was like oh my gosh it's going to be so hard. I've just noticed that the enjoyment is a lot more there and a bit more passion, especially when I get to discuss with other people. I like that concept of being able to bounce ideas off each other and learn new things so that really helped me in that way, and also seeing the opportunities that I have within uni and outside of the uni… but yeah there's so much things that I've learned from this.”   6. “So yeah the future is uncertain so I’m just saying things I think a step at a time. My buddy group leader, she told us how a student honors project, and she went into a PhD with her same supervisor and the project she is doing now, and her struggles or successes. Something like that showed me like Oh, if I were to go down a similar route I’d expect these things, so I would say, it gave me a good overview of what my future would be if I chose to do a PhD.”   7. “even just being not afraid to talk to lecturers, I was kind of apprehensive about connecting with them because they seem like people who are so high up… a bit distant, but then you realize that like oh it's okay to reach out and yeah I think it's really helped me in that way to just come out of my shell a little bit”   8. “I guess like the main thing that would come to mind would be confidence in reaching out to people in the department.” |
| Developed a sense of belonging Participants reported a greater sense of belonging to a wider group, department, or community. | - 1. “I enjoyed it, it was nice to be part of the team, and I also got to meet some new PhD students, the other fellow buddy group leaders, so that was really nice.”   2. “What I really enjoyed about the buddy program is that not only are you interacting with the students that you are assigned to but also with the other buddy group leaders, because I think that's also a unique relationship that you have and we did have a zoom together to brainstorm ideas [of] how else we can help those students but also how we can also help and be there for each other, which I think was a very important and something that should be enhanced next time around in subsequent programs.”   3. “I was looking to become more involved in the Faculty of Medical and Health Sciences, I have friends that are also doing honours with me, but I did feel a little bit more isolated this year coming into the lab and sort of felt very disjointed compared to previous years, so I wanted to have a little bit more of a support system, a little bit more of community feel within the Faculty.”   4. “Just being like feeling more comfortable in the space that I was working in was really helpful for me and really made me feel a lot safer, or like a lot more at home.”   **Improvements**   - 1. “Maybe having all the buddies of each group to connect with one another in the same way that the buddy group leaders connect. That might be helpful as well because they've also got a unique perspective, and if they had the problems with their buddy group leader, they can always talk to [the buddy group coordinators], but they can also reach out to other students potentially and that might be a different network of kinship they can be in contact with.”   2. “maybe advertising it a little differently or being able to reach a lot more people like I remember when I told my friends about it, who are also doing like an honors or postgrad, I was just surprised that they weren't a part of the buddy program just because they're a part of a lot of [other] postgrad stuff- I'm usually the person that's not a part of these things. They just didn't know about, or it came through their emails but they didn't think it was important and I was just telling them like no it's actually really fun, it's really nice, and it's not too time consuming or anything and there's no really like homework or anything you have to do – you just show up. So maybe like reaching out to more students or finding a way to do that would be nice.”   3. “The thing is, with the buddy program a lot of the seminars and workshops and things were for the buddy program or they were related to the buddy programme but it would be nice if we had exposure to other random things that were happening on campus that we could know about. So if we just had more information about other things that are happening on campus and other events, not just the ones that the buddy program chose for us.”   4. “Well, I wish, more people knew about it (laughs)” |
| Improved Coping Through COVID-19 Lockdowns Improved motivation, social supports, and ability to continue their studies despite the COVID-19 lockdowns. | - 1. “I think over zoom it was a bit tricky- that first zoom with my buddies was a real pilot on how well our relationship that was just over two sessions in person translated and whether we'd still have that relationship and engagement. And so there was a bit of nervousness there and a real perseverance, to get that relationship up and running again over zoom which I think worked really well and I also saw some problems that we were having as a group, where people were just feeling demotivated during lockdown, and so I created some writing sessions, where we all would come together over zoom and we didn't have to talk, we could just be in each other's company and work on projects.”   2. “So particularly over covid, it was because the last few buddy meetups that we did were over zoom through this most recent lockdown and so I was a little bit more isolated than I would have otherwise been, so it was nice to have people to reach out to and to also feel like I was someone who could be reached out to by other people as well, so over covid in particular gave me a sense of social support for sure.”   3. “I think the whole lockdown situation is quite difficult to get in touch with people and I think it can make you feel a little bit isolated. And so to have people there that you know you can contact for something or to show you who you should be getting in touch with or whatever has been really good throughout lockdown.”   4. “I think potentially it was more helpful with Covid, but it was different definitely because of Covid because we're having zooms and I think that was positive and since I was actually seeing people.”   5. “I think the first thing was, with it being locked down, it was nice having people that were not like my close group to just talk to, and some people who were on the outside that I could connect with, so that was nice with lockdown.”   6. “when we went into the first week of lockdown we had a session, where we were talking about how we were feeling and that was really nice, I think, where everyone was sharing tips on how to manage or keep productive so that was pretty nice”   7. “Definitely helped me with motivation … yeah especially with lockdown. Motivation’s been really hard especially when it's just you wake up and it's just another workday. And that's really all we could have done for quite a long time, but yeah so motivation and encouragement and even just learning new skills or the different tips to help me be more productive. The hardest part for me was just starting something… so it's been helpful in that way” |
| Encouraged collegiality The buddy programme was an outlet to encourage collegiality where participants were able to give back and to build towards better support systems for future students. | - 1. “It improved my sense of social support and seeing that there were so many buddy group leaders, they obviously had to volunteer because they wanted to help people. It made me feel more comfortable, knowing that there are people that you know they're just happy to answer your questions and help you out.”   2. “it's also a good way to give back to undergrad students as well and people like me have had the privilege of going through this academic pathway. And I’ve done so well, partly due to my own efforts, but also because I’ve had a lot of people around me to really help me through that journey and so it's definitely an awesome way to give back into it and ensure that you're that person for someone else.”   3. “I think one of the goals, personally was that there is some sort of support system that is established with postgrad students, so that they could approach me, even if they are having challenges, not just with academia, but in general, so I could signpost them to the right people.”   4. “I remember going to my orientation for honours and they had a panel of past students and I don't know if it was that particular year but none of them was that motivating for doing honours. They all went on about how hard it was and how difficult it was and I remember finding that quite daunting to start the year off with. So I have always signed up to do things that would rectify that because I remember feeling quite disheartened after listening to those people talk and I actually ended up really enjoying my honours year so one of the reasons, was to be more supportive for future students”   5. “I think one of the main things was that I really enjoyed my honours year and I know, there was some people who didn't- just because they felt a bit lost in the system, so I think it is important to make sure that if somebody wants support, that support is there and it's not intimidating like head of department or something like that.”   6. “I wanted for my buddy group to not feel like an awkward gathering, I wanted people to look forward to it and not feel like it was a chore. I thought that it would be the best kind of environment for me to support the students in a different way to how they are already supported. I thought that would be a challenge as well, in terms of making that environment, and that was one of the things I was looking forward to. I was just looking forward to the challenge of getting to know the people and making them feel like the buddy group was a safe space for them to talk about problems if they had any or talk about their futures, which is something I could help with.” |
